# What Do Global Health Practitioners Think about Decolonizing Global Health?

**DOI:** 10.5334/aogh.3714

**Published:** 2022-07-27

**Authors:** Madelon L. Finkel, Marleen Temmermann, Fatima Suleman, Michele Barry, Melissa Salm, Agnes Bingawaho, Peter H. Kilmarx

**Affiliations:** 1 Population Health Sciences, Weill Cornell Medicine, New York, NY, USA; 2 Dept. Obstetrics and Gynaecology, The Aga Khan University, Nairobi, Kenya; 3 Discipline of Pharmaceutical Sciences, University of Kwa Zulu-Natal, Durban, South Africa; 4 Center of Innovation in Global Health, Professor of Medicine and Tropical Diseases, Stanford University, Stanford, CA, USA; 5 Bio.Polis, Stanford University, Stanford, CA, USA; 6 The University of Global Health Equity, Kilgali, Rwanda; 7 Fogarty International Center, U.S. National Institutes of Health, Bethesda, MD, USA

**Keywords:** decolonialization, global health, global partnerships

## Abstract

The growing awareness of colonialism’s role in global health partnerships between HICs and LMICs and the associated calls for decolonization in global health has led to discussion for a paradigm shift that would lead to new ways of engagement and partnerships, as well as an acknowledgement that colonialism, racism, sexism, and capitalism contribute to inequity. While there is general agreement among those involved in global health partnerships that the current system needs to be made more equitable, suggestions for how to address the issue of decolonization vary greatly, and moving from rhetoric to reform is complicated. Based on a comprehensive (but not exhaustive) review of the literature, there are several recurring themes that should be addressed in order for the inequities in the current system to be changed. The degree to which decolonization of global health will be successful depends on how the global health community in both the HICs and LMICs move forward to discuss these issues. Specifically, as part of a paradigm shift, attention needs to be paid to creating a more equal and equitable representation of researchers in LMICs in decision-making, leadership roles, authorship, and funding allocations. There needs to be agreement in defining basic principles of best practices for global partnership, including a universal definition of ‘decolonization of global health’; the extent to which current policies allow the perpetuation of power imbalance between HICs and LMICs; a set of principles, best practices, and models for equitable sharing of funds and institutional costs among partners; a mechanism to monitor progress prospectively the equitable sharing of credits (e.g., leadership, authorship), including a set of principles, best practices, and models; and, a mechanism to monitor progress prospectively the extent to which decolonialization will contribute to strengthening institutional capacity in the LMIC institutions.

## Background

Issues of equity and power asymmetry in global partnerships are driving the current discussion of decolonizing global health (DGH). Partnerships among institutions in high-income countries (HICs), sometimes also referred to as the Global North, and low- and middle-income countries (LMICs), also referred to as the Global South, have been the bedrock of global collaborations for decades. Within the past few years, however, there has been considerable discussion among global health practitioners in both HICs and LMICs of a perceived imbalance inherent in the current system, stemming from the legacy of former colonial relationships and power inequities. The consensus is that there is a need for a reexamination of the assumptions and practices underpinning global health partnerships, including the inequitable power dynamics and neocolonialist assumptions that have been long ignored [1,2,3,4,5].

The call to recognize the colonial legacies of the past and to change the structures within which global health currently operates is fueling the global debate about diversity, equity, and inclusion. [6]. The issue, however, is not a new one. Linda Tuhiwai Smith, a Maori leader in indigenous education in New Zealand, eloquently and powerfully wrote in 1999 about scientific racism and how it has remained foundational to academic knowledge and research practices [7]. Decades later, the current system continues to perpetuate existing power imbalances as the legacies of colonialism and its lingering impact contribute to the inequity between HICs and LMICs, and that neocolonialism (e.g., the use of economic, political, cultural, or other pressures to control or influence other countries, especially former dependencies) serves to perpetuate and reinforce the colonialist paradigm of control and influence through unrecognized actions, behaviors, attitudes, and beliefs [8,9].

While there is general agreement among those engaged in global health partnerships that the current system needs to be made more equitable, suggestions for effecting change vary greatly, and moving from rhetoric to reform is complicated. For example, decolonizing global health is a complex term that may mean different things to different people. There are divergent views about the best approaches to achieving the decolonization of global health and what that might eventually look like. Moreover, there is a lack of a coherent set of principles, approaches and tools, which individually and collectively hamper the ability to arrive at consensus definition.

The growing awareness of colonialism’s role in global health partnerships between HICs and LMICs and the associated calls for decolonization in global health has led to calls for a paradigm shift that would lead to new ways of engagement and partnership, as well as an acknowledgement that colonialism, racism, sexism, and capitalism contribute to inequity. Presently, HIC institutions (academic and funders) consciously or unconsciously create a situation in which global health remains much too centered on individuals and agencies in HICs at the expense of the partners in LMICs. The HIC institution generally sets the research agenda, formulates the research questions, designs the study, obtains the funding, retains most of the overheads, conducts the analyses, presents the findings at conferences, and publishes the findings in English in journals that may be unavailable and/or unaffordable to their partner in the LMIC where the study is actually conducted.

## Survey of Global Health Leaders

In 2021, the Consortium of Universities for Global Health (CUGH) established a working group of LMIC, HIC, and indigenous voices to address the issue of DGH. To gain a more complete understanding of perceptions of equity and power imbalances in global health partnerships, a survey was conducted. The target audience included members of CUGH engaged in global health research as well as members of the African Forum for Research and Education in Health (AFREhealth), an interdisciplinary health professional grouping that seeks to improve the quality of health care in Africa through research, education, and capacity building. The survey instrument consisted of 10 closed- and open-ended questions and was drafted with input from the working group members from both HICs and LMICs. An online anonymous survey link was emailed to 170 individuals in December 2021, with a follow up reminder sent in January 2022. The following provides a summary of the quantitative and qualitative findings.

## Findings

### Quantitative Findings

Forty-four responses (response rate of 26%) were received, of which 24 (55%) were from individuals from HICs and 17 (39%) from individuals from LMICs. Three responses (7%) did not specify their country. When asked how many years they have been involved in global health partnerships, four (9.1%) reported less than five years, seven (15.9%) responded between five and nine years, 17 (38.6%) between 10 and 20 years, and 16 (36.4%) for more than 20 years.

The overwhelming majority of respondents strongly agreed (59%) or agreed (32%) with the statement: “Colonialism adversely impacts global partnerships.” Among those from HICs, two-thirds (62.5%) strongly agreed and one-quarter (25%) agreed. Among those from LMICs, half (52.9%) strongly agreed with the statement while 41.2% agreed. The findings indicate that those in HICs, compared to LMICs, feel more strongly that colonialism adversely impacts global partnerships.

When asked if they have experienced the negative effects of colonialism in their global partnership, 12 participants (27%) strongly agreed while 17 (38.6%) agreed. Among those were six from HICs (25%) and five (29.4%) from LMICs who strongly agreed with the statement. However, among those who agreed with the statement, there is a larger difference between those from HICs (41.7% who agreed) compared to those from LMICs (29.4% who agreed). One-fifth (20.8%) of those from HICs and almost one-quarter of those from LMICs (23.5%) disagreed or strongly disagreed with the statement.

For the statement: “Even though the research is being conducted at my institution, I do not feel that I have equal control over the study (e.g., study design, budget, authorship, etc.,” an equal proportion to the respondents (24%) said that they strongly agreed or agreed with the statement as disagreed or strongly disagreed. Among those from HICs, one-quarter agreed with the statement while 50% disagreed or strongly disagreed. Differences were apparent among the responses from those from LMICs. Half (52.9%) said that they strongly agreed or agreed with the statement while 29.4% said that they disagreed or strongly disagreed.

When asked about not being viewed as an equal partner in their global partnerships, 19 (43.2%) replied that they strongly agreed or agreed with the statement while 18 (40.1%) said that they disagreed or strongly disagreed. Among those in HICs, seven (29.2%) said that they agreed with the statement while over half (54.2%) disagreed or strongly disagreed. Ten individuals from LMICs (58.9%) strongly agreed or agreed that they felt that they were not viewed as an equal partner in global partnerships while 23.5% disagreed or strongly disagreed with the statement. Four individuals (16.7%) from HICs and three (17.6%) from LMICs said that they had no opinion.

### Qualitative Findings

The survey provided an opportunity for respondents to provide responses to open-ended questions to provide more personal perspectives on the topic. Respondents were asked to briefly explain why they felt that the power relationships in the collaboration are not balanced. The main theme from the LMIC respondents was that financial interests control the power in the relationship and that funders have their own agendas and enter partnerships with preconceived viewpoints. In addition, respondents felt that greater value is often attached to skill sets of HIC partners, with little if any value attached to the skill sets of LMIC partners, unless they were educated in HICs. Several respondents noted that HIC researchers have more resources and better connections to research funders and often approach LMIC researchers as inferior partners, assuming that the HIC investigator should formulate the research questions while the LMIC researchers should implement the studies. A related issue is that LMIC institutions often don’t get the same indirect (overhead) funding as HIC institutions, which perpetuates the financial and power imbalances. Table 1 summarizes key findings analyzed by HIC and LMIC status.

**Table 1:** Qualitative Responses.


QUESTION	HIC	LMICS

**Why are power relationships in the collaboration not balanced?**	*Funding equals power* *Priorities are dictated by European and US funding agencies*. *There is an unequal involvement between partners that creates an imbalance at the very start*. *LMIC partners may be too polite to challenge HIC partners*. *HIC partners do most of the work and ask for (tacit) approval from LMIC partners*. *The same people usually older white men are in control of the global health research agenda in most US universities*. Putting HIC *Institutional gain as a priority*. *Power imbalances sometimes unavoidable and not part of decolonization*.	*Financial interest is the power*. *The funders usually have their own agenda*. *Greater value attached to skill sets of Global North partners, and little value attached to skill sets of Global South partners, unless educated in the Global North*. *Researchers based in the Global North have more resources and better connections to funders* *LMIC researchers seen as somehow inferior*. *Budgets not shared and PIs are condescending and not transparent*.

**What aspects of the partnerships could be changed to make things more equitable at the individual level?**	*Increased access to resources and representation in leadership*. *Have LMIC partners interact (and be accountable) directly to funders*. *Shared leadership, faculty appointments for local leaders in countries of partnership*. *Co-PIs/co-authorship on all research and financial reimbursement/salary for local country program leaders/supervisors/educators, be at par with HICs*.	*Decisions to be made together by both parties at the planning phase of a study*. *Allow equal control over financial resources*. *Ensure research is context relevant*. *Increased funding to local organizations by international donors*. *improved capacity building and educational opportunities for local professionals*. *Provide equal opportunities to partners—share budgets, share authorships, include those with less opportunities in projects of those with more resources. Building trust and respect*.

**What aspects of partnerships could be changed to make/to ensure equity at institutional level?**	*Greater awareness of behaviors and language that reinforce colonial attitudes and practices*. *Equal engagement of partners. Mutual respect*. *Relationship building*. *Practice humility*. *Better communication*.	*Decision making should be inclusive and funds equitably distributed*. *Explicit discussion about roles and authorship* *Equitable access to information and to discussions with funders*. *More engagement on budget decision making and allocation* *Targeted hand-holding for publications*. *Expectations set at the beginning of a relationship*.

**What aspects of the partnerships could be changed to make things more equitable at the funder level?**	*Funders must be willing to have LMIC partner be the lead recipient and take full ownership*. *More career development awards for in country investigators*. *Seek to fund directly in low-income countries*. *Funders must give a consistent message about whether or not these issues are significant to them*. *Support Global South partners to lead research; don’t just ask them to add to the research*.	*Funds should be shared equally and planning should be done together. -Allow Global South partners to include agenda-setting decisions. -Funders should provide more access to all researchers in a team, not only the one based in a Global North institution. -Funders should give equal opportunities for all organizations to compete for funds and also empower organizations from LMICs*. *Directly funding each institutional partner instead of having a ‘lead’ institution and a subcontract to a second institution*. *More consultations with LMICs*.


When asked about what aspects of the partnerships could/should be changed to make things more equitable at the institutional and/or individual level, LMIC respondents made several recommendations, including the importance of building trust and respect, having explicit discussions so decisions are made transparently and jointly beginning with the project planning phase, establishing mechanisms for equitable control and distribution of financial resources, and building capacity at weaker institutions. Other suggestions from LMIC respondents focused specifically on authorship and the need to establish equal opportunities in co-authorship, especially first and last (senior) authorship.

The survey also asked about how to make things more equitable at the funder level. The LMIC respondents, in particular, recommended including LMIC partners in agenda-setting decisions so that funders may gain insight into LMIC needs and challenges. In addition, equal opportunities for competition should be given and LMIC organizations should be empowered to compete favorably. One suggestion was that each partner, HIC and LMIC, could be funded directly instead of having a ‘lead’ institution (more often than not the HIC partner) receive the funds and then subcontract to the LMIC institution.

One respondent, for whom the country income level was not stated, stated: “Treat us equally as you would treat your own scientists. Don’t say we are equal and then when a contract comes tell us to ‘Take it or leave it’!”

## Discussion

The survey helped identify areas of concern as global health practitioners address power asymmetries and inequity in partnerships. There are several limitations to this survey, including the overall low response rate, low response rate among those in LMICs, contributing to selection bias. That being said, the qualitative responses help frame the dialogue about DGH going forward. There is a general consensus of the problems in the current system that should be addressed.

## Authorship

Authorship is very important to researchers in all disciplines because it directly impacts decisions regarding hiring, tenure and promotion, and funding grants [10]. Fair distribution of authorship continues to be a concern in global health research where researchers from LMICs collaborate with researchers from high-income countries [11]. Zachariah et al. and others highlight the difficulties associated with distributing authorship in research teams conducting operational research in LMICs [12,13]. Efforts to reform the system should consider equalizing scholarly recognition; however, many various factors, including but not limited to language barriers, editorial bias (e.g., favoring prominent researchers from HICs at the expense of their LMIC collaborators), order of attribution (e.g., whose name goes first? Whose name goes last?), individually and collectively serve to influence authorship decisions [14].

Since most global health journals of international reputation are written in English, this could systematically and unjustly exclude non-English speaking researchers even if they have substantially contributed to the research project. Also, compounding the issue is the lack of guidance on authorship from medical editors; of note is that the International Committee of Medical Journal Editors (ICMJE) authorship recommendations are viewed as the leading standard in health science research, although these recommendations are not often taken into account when deciding on authorship listing [15].

## Leadership

Current leadership inequities include an over-representation of white men from HICs in global health leadership positions. Those with knowledge and experience in the project setting, individuals who have years of experience living and working in the country and speak the local language(s), often are not equally recognized for their contribution [16]. This is especially so with women in LMICs, although it is estimated that up to 75% of health workers are female [17]. This gender ratio is not reflected in the top levels of leadership in international or national health systems and global health organizations [18].

The extent to which collaborators from LMICs are listed as co-principal investigators (PIs) on grants and take the lead in overseeing the research or program project needs to be examined more fully. Given that sustainability is crucial in global health research, the LMIC co-investigator’s role is important, as this individual will be the point person to ensure that the project outlives the funding cycle. Sustainability should be the goal of global health partnerships, and not limited to the two- to three-year grant award.

## Funding

Research is heavily influenced by funding agencies, which are mainly based in HICs. While research agendas often address important areas for study, they may not reflect the interests at the host site. Further, individuals making funding decisions based on evaluation of grant submissions tend to be from HICs, and the grant is generally awarded to the HIC institution with funds then disbursed to the LMIC partner.

Global health research often depends on strong clinical, laboratory and human resource infrastructure, which often are less developed in many LMICs. Most research studies do not have the budget or funder approval to make significant investments at the host institution, perpetuating dependency and inequity. There is a need for funding agencies to develop and provide frameworks for an ethical and equitable partnership, taking into account the roles of all of the partners. Further, funders should consider selecting grant reviewers based on gender, social, geographical, and ethnic backgrounds (see Table 1 in Khan M et al.) [19].

An important stumbling block to global health equity is that research agencies in HICs often pay lower indirect or overhead costs to foreign grant recipients than they pay to their domestic grantees. A rationale for this difference is that governments have an interest in fostering and sustaining robust domestic research capacity to benefit their citizens for education, employment, and innovation over the long run. In the United States, these domestic indirect costs are called Facilities and Administrative Costs and cover grantees’ costs such as building and equipment depreciation, interest on bonds, research-related administrative costs (e.g., IRBs, animal care, and other compliance functions), security, waste disposal, utilities, libraries, and computer systems [20]. The rate is negotiated by the grantee institution. The expectation is that research institutions in the partner country should have these costs covered by their own governments. However, this is often not possible.

Many HICs provide core funding to their research institutions, but many LMICs do not, resulting in weak research infrastructure and exacerbating power imbalances between HIC and LMIC collaborators.

## Academic Initiatives in Decolonizing Global Health

There are numerous ongoing efforts at universities around the world that address the issue of decolonizing global health. Many are student-initiated and student-led. There also are calls to address the issue in the medical school curriculum (and this could/should apply to other health sciences disciplines), which are discussed in detail by Eichbaum et al. [21] and Garba et al. [22].

Academic institutions with ongoing decolonizing initiatives include (but are not limited to) the Karolinska Institute, the School of Global Health at the University of Copenhagen, the Harvard TH Chan School of Public Health, the Decolonize Global Health Working Group at the University of Edinburgh, and the Duke Decolonizing Global Health student working group at Duke University. Many other medical schools and universities are beginning to address the issue in various ways (e.g., conferences, webinars, curriculum changes, etc.). The Consortium of Universities for Global Health (CUGH) Competencies Toolkit is helpful in defining appropriate roles and competencies for trainees and professionals working toward health equity and understanding of other cultures and contexts [23,24].

## How to Move Forward?

The COVID-19 pandemic has necessitated a shift in global collaborative research. As travel from HICs was restricted and HIC partners focused more on their home-country needs, many LMIC collaborators seized the opportunity to demonstrate strong, independent leadership of their programs. These dynamics have increased momentum to decolonization and underscore the need to recalibrate power relationships to improve global health. COVID-19 travel restrictions also provided the impetus for the proliferation of virtual conferences, which enabled participants from around the world to attend without incurring the costs of visas, airfare and hotel, costs that can be prohibitive and may not be included in the project budget.

The degree to which DGH will be successful depends on how the global health community decides to move forward to discuss, define, and agree to basic principles of best practices for global partnerships. Going forward, we need to agree on the reasons why and for whom we are decolonizing global health and build consensus [25]. What is on the table is as important as who is around the table [26]. While there is no ‘right’ way to achieve this objective, the following suggestions could help guide reform.

- A universal definition of ‘decolonization of global health’ should be made with input from many different stakeholders and disciplines. Implicit is an agreement on the parameters of the definition.
- The extent to which organizations in HICs and LMICs knowingly or unknowingly perpetuate inequity is an issue that requires discussion. What are the parameters of the situation and how best can reform be enacted? To what extent do current policies allow the perpetuation of power imbalance? Answers to these questions will differ by country and site.
- There needs to be agreement on a set of principles, best practices, and models for equitable sharing of funds and institutional costs among partners.
- There needs to be agreement on the equitable sharing of credits (e.g., leadership, authorship), including a set of principles, best practices, and models.
- There needs to be agreement on ways to enhance institutional capacity in LMIC institutions.
- Funders should be brought into the dialogue on best practices for equity between LMIC and HIC grantees.

The cost of inaction is too high to ignore and preserving the status quo should not be an option. If we do not heed the calls put forward to decolonize global health, we will squander the opportunity to make the system more equitable.

## Disclaimer

The findings and conclusions in this paper are those of the authors and do not necessarily represent the views of the National Institutes of Health or other institutions.
